# Small RNA-Seq Analysis Reveals miRNA Expression of Short Distance Transportation Stress in Beef Cattle Blood

**DOI:** 10.3390/ani11102850

**Published:** 2021-09-29

**Authors:** Mingli Wu, Xiaoqin Tang, Sayed Haidar Abbas Raza, Haidong Zhao, Qi Li, Xiaohua Yi, Fayez Althobaiti, Mustafa Shukry, Xiuzhu Sun

**Affiliations:** 1 College of Animal Science and Technology, Northwest A&F University, Xianyang 712100, China; wumingli@nwafu.edu.cn (M.W.); txq@nwafu.edu.cn (X.T.); haiderraza110@nwafu.edu.cn (S.H.A.R.); 2018060160@nwafu.edu.cn (H.Z.); liqi990@nwafu.edu.cn (Q.L.); yixiaohua@nwafu.edu.cn (X.Y.); 2 Department of Biotechnology, College of Science, Taif University, P.O. Box 11099, Taif 21944, Saudi Arabia; faiz@tu.edu.sa; 3 Department of Physiology, Faculty of Veterinary Medicine, Kafrelsheikh University, Kafrelsheikh 33516, Egypt; mostafa.ataa@vet.kfs.edu.eg; 4 College of Grassland Agriculture, Northwest A&F University, Xianyang 712100, China

**Keywords:** transportation stress, beef cattle, differentially expressed miRNAs (DEMs), blood, WGCNA

## Abstract

**Simple Summary:**

In this study, three miRNA modules were identified in a cattle short-distance transportation stress model, and the turquoise module showed key miRNA sets according to their correlation with hub genes. Further, hub miRNAs were identified based on their targeting relationship with hub genes in our previous study. This finding provides the potential utility for predicting and treatment of short-distance transportation stress in beef cattle.

**Abstract:**

Transportation is a crucial phase in the beef cattle industry, and the annual losses caused by beef cattle transport stress are substantial. Because of its huge economic losses, such as lower growth rate and even death, long-distance transportation stress has attracted more attention from beef production practitioners because of its huge economic losses. Compared with the long-distance transportation stress, the short-distance transportation stress was ignored for the reason of no obvious symptoms in cattle. Our previous study showed that the disorder of B cell function could be a potential health risk after short-distance transportation. However, the transcriptome details of the changes in the cattle blood after short-distance transportation and the molecular mechanisms for the regulation of the developmental process are not clearly known. In this study, a total of 10 Qinchuan cattle were used to compare the molecular characteristics of blood before and after short-distance transportation. The miRNA-seq showed that 114 differentially expressed miRNAs (DEMs) were found (40 upregulated and 74 downregulated) between two groups before and after transportation. Furthermore, more than 90% of the miRNAs with counts of more than 10 were used to construct a co-expression network by weighted correlation network analysis (WGCNA), and four independent modules were identified. According to their relationship with 30 hub genes, the turquoise module was the key module in this study. The regulator network of hub genes and miRNAs in the turquoise module was constructed by miRNAs targeting genes predicting, and the miRNAs had targeting sites within hub genes that could be identified as hub-miRNAs. Further, it showed that CD40 and ITPKB had the same targeting miRNAs (miR-339a/b), and the newly discovered hub miRNAs filled the gaps in our previous study about the relationship between hub genes in short-distance transportation stress and provided the potential utility for predicting and treatment of short-distance transportation stress in beef cattle.

## 1. Introduction

With the improvement of social productivity, animal welfare issues have become increasingly prominent. Animal welfare not only affects the production efficiency of animal husbandry, but also affects human health seriously. Transportation stress is an important link in animal production, involving the economic benefit and welfare of animal production [1]. Transportation stress can be defined as the change of environment significantly that disturbs body homeostasis. Compared with the transportation stress process of other livestock, beef cattle are paid more attention because of the particularity of their digestive system, especially the rumen digestive function. Transportation stress has brought huge economic losses to the beef cattle industry, which could be divided into long-distance transportation stress and short-distance transportation stress [2,3]. On the one hand, long-distance transportation stress has attracted more attention from beef production practitioners, because it can slow down the growth rate and even cause death of beef cattle [4,5]. The mechanism of long-distance transportation was interpreted, including hormone, amino acids, glucose, lipid, cholesterol, immunocyte, gastrointestinal microbiota, nasopharyngeal microbiota, and carcass quality [6,7,8,9,10]. On the other hand, most cattle were normal apparently after short-distance transportation, but could have potential health risks. The mechanism of short-distance transportation was still poorly understood compared with long-distance transportation. Our previous study showed that the disorder of B cell differentiation, proliferation, survival, and apoptosis were the potential molecular mechanisms in short-distance transportation stress [11]. It suggested that beef cattle are susceptible to the attack of pathogenic bacteria after short-distance transportation. Considering the production efficiency and animal welfare of beef cattle, attention should be paid to the transportation stress of short-distance transport of beef cattle. According to the results of transcriptome analysis, three potential molecular markers genes were identified in 30 hub genes, but the regulatory network of hub genes is still unknown. Noncoding RNAs participate in the restoration of cellular homeostasis or adaptation to environmental conditions through changes in the gene expression programs. As an important factor of noncoding RNAs, microRNAs (miRNAs) play key roles in the regulation of cellular homeostasis in eukaryotic organisms. While the uses of miRNA in the diagnosis of transportation stress and its mitigations are in their early phases, successful miRNA-based therapeutics have been established in essential exploration. The transcriptome details of what changes occur in the cattle blood after short-distance transportation and the molecular mechanisms for the regulation of the developmental process are not clearly known. 

This study aimed to explore the change in blood transcriptome of miRNAs after short-distance transportation, thereby updating the theoretical basis for the diagnosis of Beef cattle transport stress syndrome (TSSBC) and providing targeting sites of potential miRNA-based therapeutics for the management of beef cattle production and safeguarding of animal welfare.

## 2. Materials and Methods

### 2.1. Animal Model of Short-Distance Transport Stress

Ten healthy, unrelated female beef cattle (3~4 years old) were used to construct the short-distance transportation stress model. Before transportation, the cattle were kept in loose housing conditions and fed on a total mixed ration (TMR). The beef cattle were deprived of food and water that day until after transportation. Blood samples were collected from each cattle before transportation, marked group B. After transportation, blood samples were collected immediately and marked group A. Group B was control group. The transportation route was: from Qinbao Cattle Industry Co., Ltd in Yangling county, Shaanxi province to Qinbao Cattle Industry Co., Ltd in Qishan county, Shaanxi province. The average transportation density was 1.28 m^2^/head, which was similar to the production process. The transportation distance was 70 km, and the vehicle was a single-layer Foton cart with a maximum speed of 60 km/h and an average speed of 30 km/h. The interval time between the two blood samples collection was 6–8 h. The temperature ranged from 26~32 °C, and the humidity was 70% [11]. 

### 2.2. RNA Extraction, sRNA Library Construction and Sequencing

Twenty blood samples were collected in sodium heparin anticoagulant containing tubes. ACK lysis buffer lysed erythrocytes, karyocytes were harvested following centrifugation at 4000 rpm for 6 min at 4 °C, and then transferred into Eppendorf tubes containing TRIzol, and finally stored in liquid nitrogen. Total RNA was isolated using the RNAiso Plus kit (Takara, Tokyo, Japan). RNA concentration and quality were evaluated using Qubit2.0 RNA detection kit (Life, California, USA) and nanodrop 1000 (Thermo, Massachusetts, USA). Considering the quality of RNA, fourteen miRNA cDNA libraries were built in this study. The steps of miRNA cDNA library construction were the following: Ligated with 3′ and 5′ adapters (Illumina, San Diego, CA, USA) using T4 ligase (New England Biolabs), purified RNA was reverse-transcribed into the first strand cDNA and amplified by PCR using primers complementary to the adaptor sequences. The final small RNA sequencing library was prepared by purified the nucleotide fractions at 140~150 bp length. After then, each library was loaded into a single Illumina Hiseq (Illumina, San Diego, USA) lane with 75 bp single-end sequencing. 

### 2.3. Primer Design and qPCR

The primers of miRNA were designed by tailing reaction on Sangon online software (https://www.sangon.com/newPrimerDesign, accessed on 20 August 2020) (Table 1). miRNA First Strand cDNA Synthesis (Tailing Reaction) (Sangon, Shanghai, China) was used for reverse transcription of RNA to cDNA. qPCR was carried out in Y480 Real-Time PCR Detection System (Roche, Basel, Switzerland) utilizing SYBR green detection (Takara Bio, Tokyo, Japan). The amplification protocol was as follows: 95 °C for 30 s, followed by 50 cycles of 95 °C 10 s, and 60 °C for 30 s. Melt curve analysis was performed between 55 and 95 °C, with a 0.5 °C increment every 5 s. Samples were run in triplicate. Forward primers were listed in Table 1 and reverse primers were provided in the cDNA Synthesis kit. U6 was used as the reference gene, its primers were kept secret and also provided in cDNA Synthesis kit. All expression levels were normalized to that of U6 and quantified using the 2-ΔΔCt method [12]. Twelve differentially expressed miRNA (DEMs) (fold-change > 2 and false discovery rate (FDR) < 0.05) were randomly chosen for verification by qPCR.

### 2.4. Small RNA-Seq Analysis and Statistics

Fast QC (http://www.bioinformatics.babraham.ac.uk/projects/fastqc/, accessed on 23 August 2020) was used for quality assessment [13]. Trimmomatic (http://www.usadellab.org/cms/?page=trimmomatic, accessed on 23 August 2020) was used to filter the reads containing adapter, reads containing poly-N, and low-quality reads [14]. All reads were mapped to sRNA, tRNA, snRNA, and snoRNA from Rfam database and filtered using blastn. Then all the reads were mapped to the reference genome (Bos taurus ARS-UCD1.2), the reads mapped to exon and not mapped to intron were filtered. The known miRNA sequences was obtained from the mirbase database (http://www.mirbase.org/ftp.shtml, accessed on 23 August 2020), and mirDeep2 software(V2.0.0.8) (https://www.mdc-berlin.de/8551903/en/, accessed on 24 August 2020) was used for novel miRNA prediction, miRNA secondary structure prediction, and miRNA quantification. DEMs were determined by edgeR [15], and satisfied the fold-change > 2 and FDR < 0.05. Targeting genes of DEMs were predicted by miranda (http://34.236.212.39/microrna/getDownloads.do, accessed on 24 August 2020). Gene ontology (GO) enrichment analysis and Kyoto encyclopedia of genes and genomes (KEGG; https://www.kegg.jp/, accessed on 28 August 2020) pathway enrichment analysis were performed using DAVID (http://david.abcc.ncifcrf.gov/, accessed on 28 August 2020), and satisfied the condition that FDR < 0.05 [16]. For weighted correlation network analysis (WGCNA) [17], more than 90% of the miRNAs with counts more than 10 were used to construct a co-expression network. WGCNA parameters were as follows: power = 6, minModuleSize = 30, networkType = “signed”, corType = “Pearson”, TOMType = “signed”, mergeCutHeight = 0.25. The relationship between hub genes [11] in the turquoise module and different modules of miRNA was constructed by correlation analysis. The relationship between hub genes and their targeting miRNA in key miRNA module was constructed by miranda.

## 3. Results

### 3.1. Preliminary Analysis of RNA-Seq Data and Verification by qPCR

**Figure 1:**
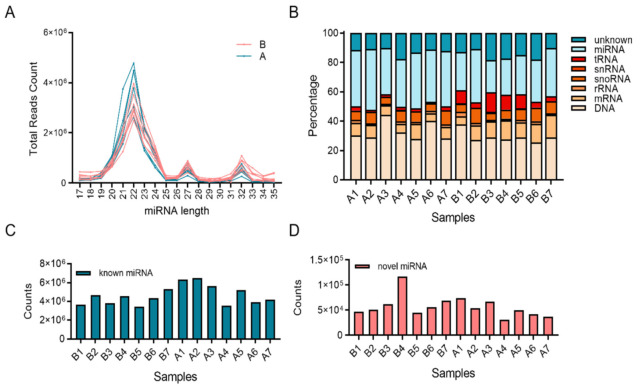
Summary of the small RNAs (sRNAs) sequencing data and the distribution of sRNAs. (**A**) Length distribution of sRNAs between before and after transportation. (**B**) Annotation distribution of sRNAs between before and after transportation. (**C**) Known miRNA reads statistics in 14 samples. (**D**) Novel miRNA reads statistics in 14 samples.

**Figure 2:**
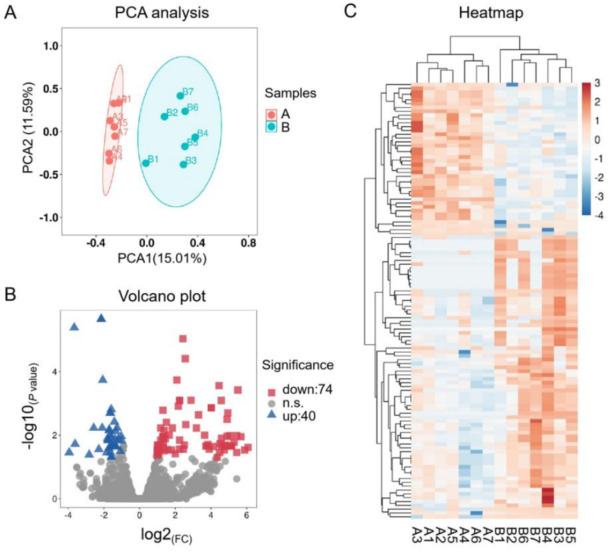
PCA and differentially expressed miRNAs (DEMs) before and after transportation. (**A**) PCA analysis of before and after transportation. (**B**) Volcano plot of DEMs between before and after transportation. (**C**) Heatmap of DEMs between before and after transportation.

**Figure 3:**
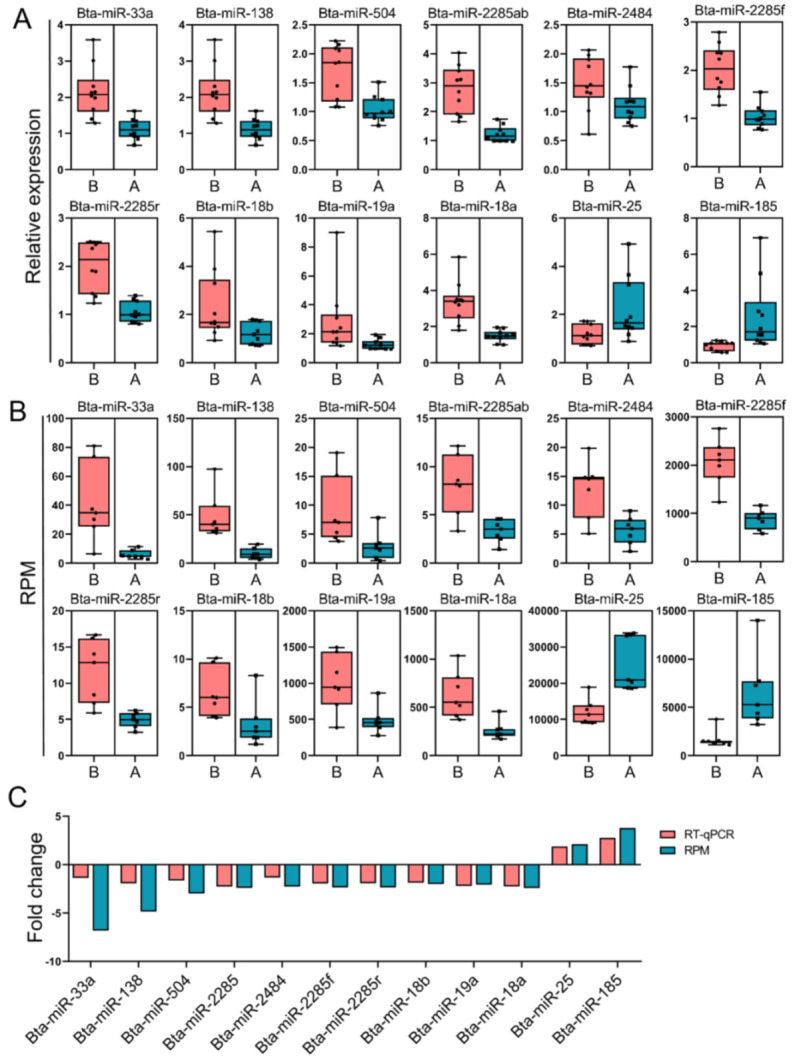
Verification of the differentially expressed miRNAs (DEMs) by PCR. (**A**) The relative expression levels of the selected DEMs by qPCR. (**B**) Log (transcript count per million [RPM]) of DEMs by RNA-seq. (**C**) Fold-change of DEM levels for both log (RPM) and qPCR.

Fourteen libraries representing seven animals in each group (before and after transportation) were prepared from total leukocyte miRNA, and group B was the reference group. The range of total reads counts for the 14 samples were 11,003,312~18,920,754 (Table 2). The range of clean reads counts for the 14 samples were 9,269,728~16,096,861 (Table 2). The range of mapped ratio for the 14 samples were 79.41~89.20 (Table 2). The range of average reads length were 22.50~24.30, most of reads distributed from 19 to 25 bp (Figure 1A). For each sample, the Q20 base ratio was >97.98%, and the Q30 base ratio was >96.82% (Table 2). The percentage of miRNA in total reads was between 21.84~41.12% (Figure 1B). Known miRNA were more than 90% of all miRNAs, the rest were novel miRNA (Figure 1C,D). Principal component analysis (PCA) showed that 14 samples were classified into two groups (group B and group A; Figure 2A). There were 114 DEMs (fold-change > 2 and FDR < 0.05) between group B and group A (Table S1), including 40 upregulated miRNAs and 74 downregulated miRNAs (Figure 2B). All the DEMs were shown in the heatmap (Figure 2C). Twelve DEMs (fold-change > 2 and FDR < 0.05) were randomly chosen for verification by qPCR. All the DEMs showed the same trend as the RNA-seq data (Figure 3).

### 3.2. Enrichment of DEMs between before and after Transportation

**Figure 4:**
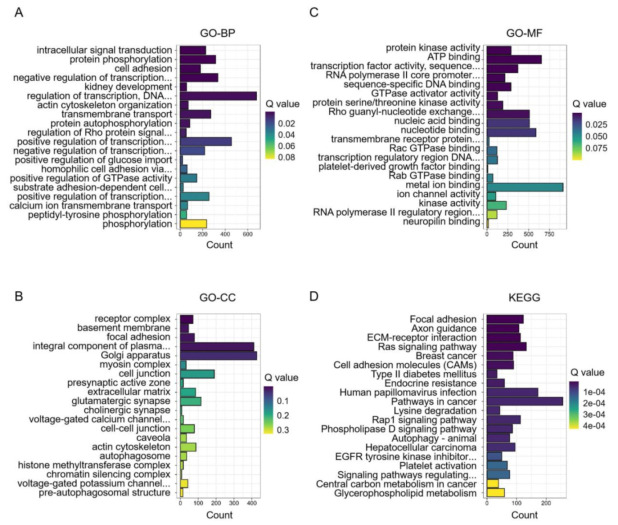
Enrichment analysis of differentially expressed miRNAs (DEMs) before and after transportation. (**A**) GO-BP analysis. (**B**) GO-MF analysis. (**C**) GO-CC analysis. (**D**) KEGG analysis.

To identify key differences between before and after transportation, GO and KEGG enrichment were performed to determine DEMs’ function (Tables S2 and S3). The biological process (BP) of GO terms showed DEMs those are involved in intracellular signal transduction, protein phosphorylation, cell adhesion, etc. (Figure 4A). The cellular component (CC) of GO terms showed DEMs those are involved in receptor complex, basement membrane, focal adhesion, etc. (Figure 4B). The molecular function (MF) of GO terms showed DEMs those are involved in protein kinase activity, ATP binding, transcription factor activity, sequence-specific DNA binding, etc. (Figure 4C). The KEGG analysis showed 104 pathways were significantly enriched (FDR < 0.05). The pathways were mainly focal adhesion, axon guidance, ECM–receptor interaction, etc. (Figure 4D).

### 3.3. Co-Expression Analysis and Their Relationship with Hub Genes

**Figure 5:**
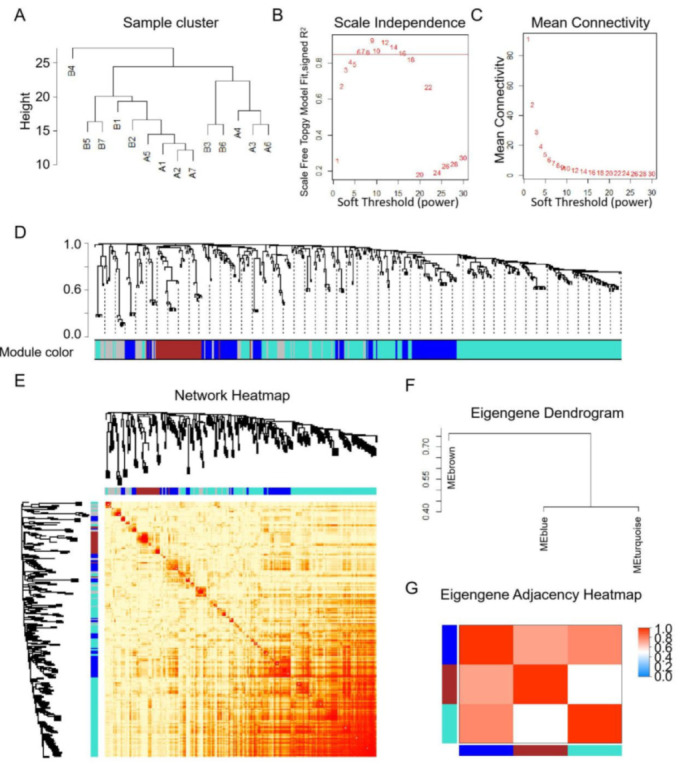
WGCNA of miRNAs before and after transportation. (**A**) Samples clustering analysis of before and after transportation. (**B**) Scale independence of co-expression. (**C**) Mean connectivity of co-expression. (**D**) Cluster dendrogram of miRNAs. (**E**) Network heatmap of DEMs. (**F**) Eigengene dendrogram of modules. (**G**) Eigengene adjacency heatmap.

**Figure 6:**
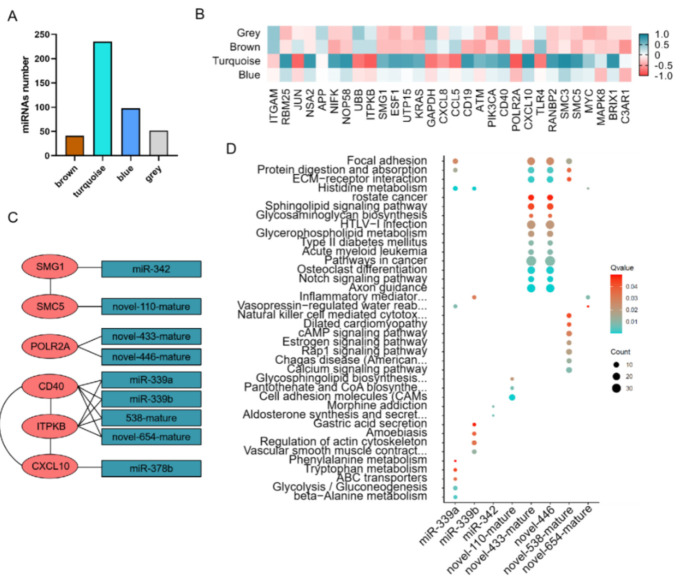
The relationship between hub genes in turquoise module and miRNA in different modules by WGCNA. (**A**) The number of miRNA in different modules. (**B**) Heatmap of the correlation between hub genes and miRNA in different modules. (**C**) Targeting relationship between hub genes and miRNA in different modules. (**D**) KEGG analysis of miRNA target genes.

All the miRNAs with their counts greater than 10 in more than 90% samples were used to construct a co-expression network. After the samples cluster, all the samples were used to construct a co-expression network (Figure 5A). The first power number when correction index more than 0.85 were selected for the next analysis (power = 6, Figure 5B). When power = 6, the mean connectivity was less than 10 and suited for the next analysis (Figure 5C). According to the parameters list in 2.4 RNA-seq analysis and statistics, three modules (blue, brown, and turquoise) were identified in 14 samples, which respectively have 98, 42, and 235 miRNAs within (Table S4 and Figure 6A). The results of network heatmap indicated a significant difference among modules (Figure 5D–F). The correlation of hub genes and miRNA modules showed that the turquoise module was the key module in the short-distance transportation stress model (Figure 6B). According to the relationship between miRNAs in turquoise module and hub genes in our previous study, we found CD40 and ITPKB have the same targeting miRNAs (Figure 6C).

## 4. Discussion

Transportation stress is closely related to animal welfare and animal productivity. Transportation stress is essentially a phenomenon of shock to the homeostasis of the organism. It was found that cortisol levels were higher after the transportation than before in cattle [1]. In order to reduce the damage to animals caused by transport stress, it is of great significance to construct transcriptional regulatory networks based on transport stress. miRNA may be involved in post-transcriptional regulation and can explain part of the gene expression that cannot be explained by transcriptional level. Our previous study did not reveal the hematological molecular mechanism of transportation stress fully, revealing the expression pattern of miRNA after transportation could elucidate the internal relationships between hub genes and enrich the regulation mechanism of transcriptional transport stress was enriched at the epigenetic level. Because there was no obvious symptom after short-distance transportation, it had not been sufficiently touched upon as they are supposed to. Based on the model of short-distance transportation stress in beef cattle, the purpose of this study was to reveal the role of miRNA in short-distance transportation stress in cattle blood, in order to provide a theoretical basis and potential therapeutic target for the prevention and treatment of beef cattle short-distance transportation stress.

The results of Figure 1 showed that the reads length was enriched from 19~25 bp and 21%~41% of the reads were miRNAs. Most of reads were known miRNAs and part of novel miRNAs were predicted in this study. Through qPCR verification, 12 randomly selected miRNAs have the same tendency as miRNA sequencing, which showed sequencing data has certain credibility in this study. GO and KEGG enrichment were used to identify the function of miRNA between before and after transportation, and the pathways related to energy supply were enriched, such as the insulin signaling pathway, type II diabetes mellitus, and sphingolipid signaling pathway. It indicated that the energy supply was a key factor in short-distance transportation stress, and confirmed the results of our previous study about mRNA expression pattern in short-distance transportation stress. The reason for transportation stress was complex, including temperature, wind, rain, hunger, thirst, crowding, shock, turbulence, social interaction, feed change, physical exertion, environmental change, and invasion of pathogenic bacteria, and part of miRNA were confirmed in recent studies [18,19,20,21]. miRNA sequencing in chronic heat stress showed that miR-199b was highly expressed and had the same expression tendency in this study [22]. miR-2284 families were also verified in circulating-miRNA expression in lactating Holstein cows under summer heat stress [23]. It suggested that miR-199b and miR-2284 families were related to heat stress response. The conditions of transportation stress were unique and unrepeatable, and the molecular mechanism of transportation stress was incomplete, especially in short-distance transportation stress. Numerous studies have begun to address the details of transportation stress, such as fasting and management [24,25]. Their focus was on hormone and mRNA expression, and the part of miRNA expression was lacking. The transcription factor NF-κB is involved in the inflammatory response and the process of immunity [26]. It has been reported that there is a direct link between stress-induced NF-κB activation and the upregulation of some miRNA [27]. It suggested that miRNA plays an important role in immune response during stress. In addition, studies have shown that stress conditions can change the function of miRNA and the expression of mRNA targets. In addition to the expression levels of miRNA and mRNA targets, the function of miRNA depends on the miRNA that has a similar target near the target [28]. It was with regret that no hub miRNA was identified through GO and KEGG enrichment analysis, and then WGCNA was used to identify hub miRNAs in short-distance transportation stress. Three modules were identified in the short-distance transportation stress model. Based on their correlation of hub genes set in our previous study, the turquoise module was considered as a key module. Combined with the targeting relationship between hub genes and miRNAs in turquoise module, nine miRNAs were considered as hub miRNAs in short-distance transportation stress. miR-339a, miR-339b, miR-538-mature, and novel-654 mature all have target sites of CD40 and ITPKB gene, and this revealed the regulatory network of hub genes at the epigenetic level. The CD40 was a specific B cell surface marker and was associated with the differentiation of B cells to memory B cells, and ITPKB have an important essential function of survival of naïve mature B cells, which indicated that the miRNA could affect the immune response of cattle by CD40 and ITPKB in transport stress [29,30,31,32,33]. These newly discovered hub miRNAs fill the gaps in our previous study about the relationship between hub genes in short-distance transportation stress. Despite that limitation, we believe our findings have the potential utility for predicting and treatment of short-distance transportation stress. Interestingly, previous studies have also identified the miRNAs that we recognized as influential on short-distance transportation stress. Further investigations with larger numbers and different conditions of transportation stress models are appropriate to validate the application of using these biomarkers and the proposed scoring mechanism.

## 5. Conclusions

In this study, 10 Qinchuan cattle were used to compare the changes of blood molecular characteristics before and after in short-distance transportation. One hundred and fourteen differentially expressed miRNAs were identified by miRNA-seq and four key modules were identified by WGCNA. Combined with the targeting relationship between miRNA and hub gene in previous research, we further screened four significant miRNAs, which provided a certain basis for the diagnosis of short-distance transport stress.

## Figures and Tables

**Table 1:** List of the primers used in this study.

Gene Symbol	Sequence (5′-3′)	Accession
bta-miR-33a-F	CGGTGCATTGTAGTTGCATTGCA	NR_031206.1
bta-miR-138-F	AGCTGGTGTTGTGAATCAGGC	NR_030863.1
bta-miR-504-F	AGACCCTGGTCTGCACTC	NR_037357.1
bta-miR-2285ab-F	CGCGAAAACCTGAATGAACTTCTTGG	NR_107841.1
bta-miR-2484-F	CGGAGCTATGATGACTTTGATTGCAT	NR_031273.1
bta-miR-2285f-F	CGCGAAAACCTGAATGAACTTTTTGG	NR_107730.1
bta-miR-2285r-F	CGCAGAAACCTGGATGAACTTTTTGG	NR_107819.1
bta-miR-18b-F	CGCTAAGGTGCATCTAGTGCAGTTA	NR_031266.1
bta-miR-19a-F	CGCTGTGCAAATCTATGCAAAACTGA	NR_030794.1
bta-miR-18a-F	CGCTAAGGTGCATCTAGTGCAGATA	NR_030892.1
bta-miR-25-F	CATTGCACTTGTCTCGGTCTGA	NR_030941.1
bta-miR-185-F	TGGAGAGAAAGGCAGTTCCTGA	NR_031178.1

**Table 2:** Quality control summary of miRNA-seq data.

Samples	Total Reads	Clean Reads (%)	Average Length	Q20	Q30	GC Percentage
B1	14,094,654	11,203,765 (79.49)	23.58	97.98%	96.82%	48.15%
B2	13,007,575	11,220,138 (86.26)	23.38	98.06%	97.07%	46.54%
B3	17,654,231	14,020,104 (79.41)	24.30	98.11%	97.13%	46.59%
B4	18,920,754	15,296,531 (80.85)	24.14	98.13%	97.16%	45.83%
B5	12,983,637	10,692,413 (82.35)	24.29	98.00%	96.92%	46.88%
B6	15,186,129	12,341,422 (81.27)	23.67	98.11%	97.15%	45.81%
B7	16,294,446	14,284,501 (87.66)	23.38	98.17%	97.21%	44.80%
A1	16,721,024	14,557,475 (87.06)	23.08	98.14%	97.16%	45.85%
A2	15,938,206	14,048,794 (88.15)	23.01	98.12%	97.15%	45.79%
A3	18,044,894	16,096,861 (89.20)	22.63	98.02%	97.03%	45.50%
A4	11,003,312	9,269,728 (84.24)	23.22	98.03%	97.04%	47.14%
A5	13,898,637	12,190,422 (87.71)	23.24	98.09%	97.07%	45.90%
A6	11,140,389	9,773,101 (87.73)	22.50	98.07%	97.10%	45.66%
A7	11,160,148	9,815,072 (87.95)	23.36	98.08%	97.08%	46.24%

Notes: B: before transportation; A: after transportation.

## Data Availability

The high-throughput sequencing data of the small RNA-seq have been saved in the NCBI Sequence Reading Archive (https://www.ncbi.nlm.nih.gov/sra/), with the accession number PRJNA764198 (SRR15944129-SRR15944142).

## Supplementary Materials

The following are available online at https://www.mdpi.com/article/10.3390/ani11102850/s1, Table S1. The differential expression miRNAs (DEMs) list. Table S2. The GO analysis of the DEMs. Table S3. The KEGG analysis of the DEMs. Table S4. Module genes of WGCNA.
